# The impact of social distancing and public behavior changes on COVID-19 transmission dynamics in the Republic of Korea

**DOI:** 10.1371/journal.pone.0238684

**Published:** 2020-09-24

**Authors:** Soyoung Kim, Youngsuk Ko, Yae-Jean Kim, Eunok Jung

**Affiliations:** 1 Department of Mathematics, Konkuk University, Seoul, Republic of Korea; 2 Department of Pediatrics, Samsung Medical Center, Sungkyunkwan University School of Medicine, Seoul, Republic of Korea; Centers for Disease Control and Prevention, UNITED STATES

## Abstract

**Background:**

In the Republic of Korea (ROK), social distancing and public behavior changes mitigated COVID-19 spread. However, a second wave of the epidemic is expected in the fall if neither vaccine nor antiviral drugs become available. This study investigated the impact of non-pharmaceutical measures on short- and long-term outbreak dynamics.

**Methods:**

A mathematical model based on Susceptible-Exposed-Infectious-Recovered model is developed considering isolated and behavior-changed groups. Using the least-squares fitting method, transmission and behavior change rates were estimated using cases reported from February 16 to April 20, 2020.

**Findings:**

The estimated transmission rate of COVID-19 was 4·6180 and behavior change rate was 2·6044. The model predicted the number of new cases to continuously decrease, with less than one case expected after May 6, 2020. Concurrently, a 25% reduction in behavioral changes during the outbreak would increase the case count by 60,000, resulting in 4,000 cases at maximum, exceeding the medical system’s capacity. As behavioral restrictions are eased, local transmission will likely increase, with forecasted second wave peak in October 2020.

**Interpretation:**

Social distancing and public behavior changes have curbed the spread of COVID-19 in the ROK. Mathematical modeling demonstrates the importance of these measures in reducing and delaying outbreaks. Nevertheless, non-pharmaceutical interventions cannot eliminate the disease. In the future, vaccines and antiviral treatments combined with social distancing and public behavior changes will be paramount to ending COVID-19 epidemic.

## Introduction

The ongoing outbreak of novel coronavirus disease-2019 (COVID-19) is a global threat to public health. The World Health Organization (WHO) declared a Public Health Emergency of International Concern [1] on January 3, 2020 and proclaimed COVID-19 outbreak a pandemic on March 12, 2020. As of April 19, 2020, a total of 2,241,359 confirmed cases, including 152,551 deaths due to COVID-19 have been reported by the WHO [2].

In the Republic of Korea (ROK), the index patient of COVID-19 was reported on January 20 who came from Wuhan, China. During the early stages of the epidemic, extensive contact tracing and immediate isolation of the confirmed cases was implemented by the Korea Center for Disease Control and Prevention (KCDC). However, on February 16, the government reported the first case without an epidemiological link. The number of cases subsequently exploded, leading the KCDC to announce COVID-19 a high-level concern (red alert) on February 23, 2020 [3].

To curb the local transmission, the government has strongly promoted non-pharmaceutical interventions, including screening of individuals at high risk of infection who had close contact with a confirmed case, immediate isolation of all confirmed cases, three times announcements of school closure, and a public campaign for social distancing and behavior change. Korean citizens were instructed to prevent local transmission by practicing good personal hygiene such as wearing a mask, washing hands, and refraining from participating in gatherings. Thanks to these non-pharmaceutical measures, the number of new confirmed cases reported in the past week was below 30. Non-pharmaceutical interventions can delay a peak of an epidemic and mitigate disease spread; however, by themselves, they cannot eliminate the epidemic. Moreover, prolonged social distancing has high socio-economic costs.

Mathematical modeling and simulation studies have provided an evidence base for public health authorities to implement prevention and mitigation strategies, while allowing to forecast dynamics of an emerging epidemic. Several recent modeling studies have focused on COVID-19 spread. For example, a study by Kucharski et al., using stochastic modeling of data from Wuhan, China, and international cases has shown that the median daily reproduction number has declined from 2.35 to 1.05 once travel restrictions had been introduced [4]. Moreover, they have shown the importance of immediate response by an explosive increment of human-to-human infection probability. Meanwhile, Prem et al. have modeled location-specific contact pattern in Wuhan, China. They assessed the impact of interventions such as school closures or workplace distancing, indicating that social distancing can effectively reduce the speed and size of an outbreak [5].

The present study examined the impact of non-pharmaceutical interventions on short- and long-term epidemic dynamics using a Susceptible-Exposed-Infectious-Recovered (SEIR) model considering isolated and behavior-changed groups in the ROK. Using mathematical modelling, we assessed the effectiveness of the ROK government and citizens’ response to COVID-19 epidemic that included social distancing and public behavior change.

The aim of this study was to estimate the COVID-19 transmission rate in the ROK, and the impact of social distancing and public behavior change on outbreak dynamics, including the peak and size of the first wave of the epidemic. The limitations of non-pharmaceutical interventions and the risk of a long-term outbreak reoccurrence are discussed as well as the benefits of short-term disease spread mitigation.

## Methods

### Data sources

Daily confirmed cases and case-fatality rates were obtained from the KCDC press releases (last retrieved on April 20) [6]. Data on the total population were retrieved from the Korean Statistical Information Service [7]. All data are publicly available, and All data were fully anonymized before we accessed them. Neither ethical approval of an institutional review board nor written informed consent we required.

### Model structure

**Fig 1:**
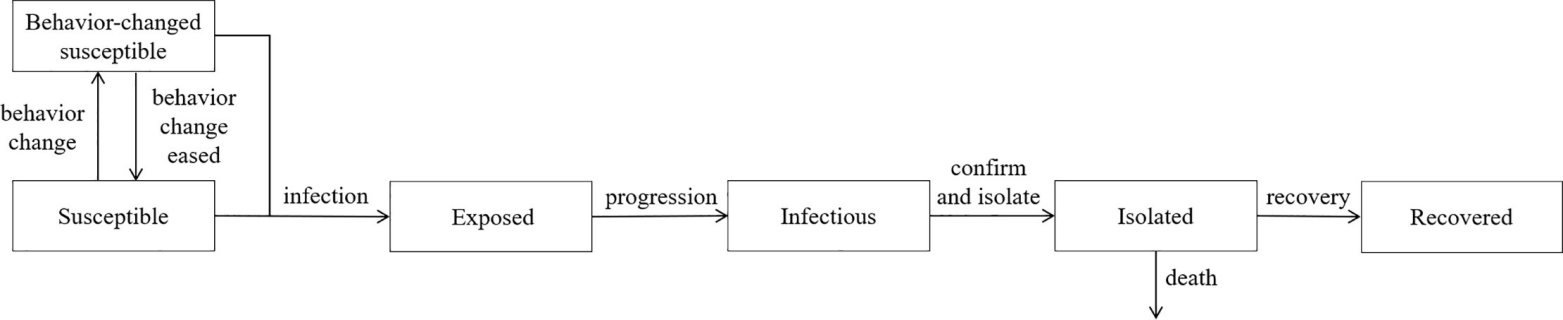
Flow diagram of COVID-19 transmission dynamics.

A dynamic model of COVID-19 transmission was developed based on a deterministic compartment model. The total population was divided into six classes: susceptible, behavior-changed susceptible, exposed, infectious, isolated, and recovered individuals (Fig 1).

Individuals in the susceptible group become infected and move to the exposed group with the incubation of the virus. Exposed individuals subsequently move to the infectious group. As the number of confirmed and isolated cases increases, susceptible individuals change their behavior and move to the behavior-changed susceptible group where the transmission rate is lower [8]. Behavior changes in this model account for all social distancing interventions such as avoiding mass gathering, and maintaining personal distance and hygiene habits. School closures and remote working were also accounted for among the behavioral changes considered. Exposed individuals move to the infectious group after the incubation period; infectious individuals are isolated after laboratory confirmation. In this study, infectious individuals were assumed to be isolated immediately after confirmation. The isolated individuals are removed through either COVID-19 induced death or recovery. This study did not account for births or deaths. Fig 1 presents a diagram capturing COVID-19 transmission dynamics.

Transmission rate among behavior-changed susceptible individuals relative to that of the general susceptible individuals was reduced by 1/50.

The progression rate was set to 1/4.1, using the mean incubation period of the virus [9]. The mean period from symptom onset to case confirmation and isolation was assumed to be 4 days, yielding the confirmation and isolation rate of 1/4 [10]. The mean period from confirmation to recovery was set as 14 days, which was the average period from confirmation to discharge among 16 patients. The recovery rate was set as 1/14. The case-fatality rate was 2.21%, based on the estimate published in the KCDC press release from April 20 [6]. Details of the mathematical model are provided in S1 File.

The model was based on the estimates reported in the KCDC daily media briefing. Local transmission dynamics modellings in the present study started on February 16, which was when the first local transmission case was confirmed. The first 28 cases before February 16 were not included in this study.

## Results

### Parameter estimation

**Fig 2:**
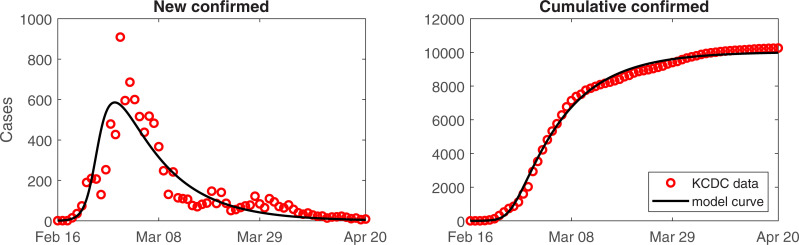
The number of confirmed reported cases and the corresponding best-fit curve: Daily (left) and cumulative (right) number of confirmed cases.

The transmission rates of COVID-19 and behavior change rates were estimated by minimizing the error between the cumulative number of confirmed cases and model curve. Fig 2 presents the number of confirmed cases (circle) and corresponding model curve (black curve) from February 16 to April 20, 2020. To consider local transmission, 376 cases confirmed at an airport were not considered in this model. There was a good fit between KCDC reported data and model curve. The estimated transmission rate of COVID-19 was 4.6180 and behavior change rate was 2.6044. Basic Reproductive number (*R*_0_) indicates the expected number of secondary cases from a single infectious individual during his/her infectious period in a susceptible population. (Effective) reproductive number (*R*) is the reduced basic reproductive number by nonpharmaceutical intervention strategy implemented by the government and it can be calculated as the product of transmission rate and averaged infectious period (1/*α*). In this work, the susceptible population is divided into two groups (susceptible and behavior changed susceptible) and each population has different transmission rate (*β* and *δβ*). Thus, the reproductive number is expressed as time dependent function $\frac{\beta }{\alpha }(S(t)+\delta S_{F}(t))\frac{1}{N}$, and we calculated the mean value of the function which is 2.2370.

### Social distancing effect on short-term epidemic dynamics

**Fig 3:**
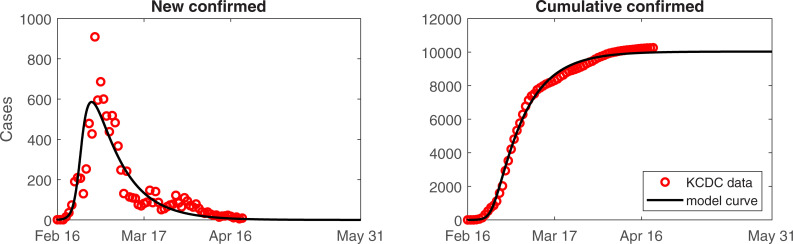
Predicted number of cases from the model until mid-May. Daily (left) and cumulative (right) number of confirmed cases.

Social distancing, increased diagnostic capacity, and extensive contact tracing and quarantine control have allowed to flatten the epidemic curve. As shown in Fig 3, social distancing and public behavior changes were effective at mitigating the spread of the epidemic.

The model predicts that the number of new confirmed cases will continue to decrease between now and May 6, 2020, when the expected daily new case is less than one.

The model-based estimates indicate that many susceptible individuals adhere to social distancing. Analysis of the effect of public behavior changes on the number of confirmed cases revealed. If behavior change reduced by approximately 75%, approximately 4,000 more cases would have occurred. Moreover, a 25% reduction in public behavior changes would result in 60,000 more cases which is equivalent to > 4,000 new cases per day, which is beyond the capacity of the medical system in the ROK.

### Social distancing effect on long-term epidemic dynamics

We assumed that behavior-changed susceptible individuals resume their normal lives and return to the general susceptible group while the number of recovered and returned susceptible individuals increases. The period of interest for this simulation was extended until December 31, 2020, to observe long-term epidemic dynamics.

**Fig 4:**
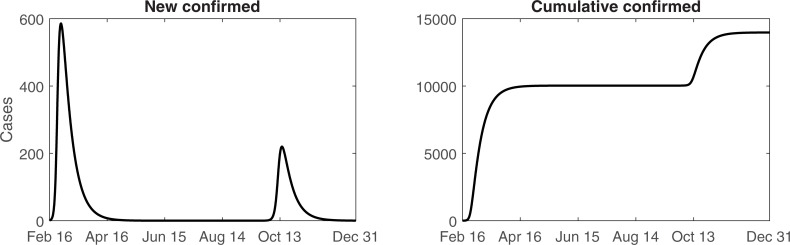
Expected number of cases from the model until the end of 2020. Daily (left) and cumulative (right) number of confirmed cases.

Although the model estimates the daily number of new confirmed cases to be <1 until September 2020, the risk of sporadic cases every few days remains. If public behavioral changes are maintained, the second wave might be prevented. However, once these measures are reduced, the rate of local transmission will again increase, resulting in the second wave epidemic peak in October 2020 (Fig 4). The second peak and number of cases in the second wave might be lower compared to the first wave. These estimates are based on the assumption that susceptible individuals return to their behavior change as new confirmed cases are reported.

## Discussion

The ROK government has introduced measures to prevent COVID-19 transmission. These measures included screening and quarantining of confirmed cases and close contacts, school closures, recommendations on strict personal hygiene and refraining from non-essential gatherings, and frequent disinfecting and ventilating of shared spaces. The combination of these policies and public cooperation resulted in a decreased number of cases. These social distancing efforts are reflected in the model in the behavior-changed susceptible category. The disease transmission rate and behavior change rate was estimated as 4.6180 and 2.6044, respectively. The mean effective reproduction number from February 16 to May 6 was estimated as 2.2370. This modeling study has shown that the ROK government and citizens have successfully responded to the spread of COVID-19 using only non-pharmaceutical interventions such as social distancing and public behavior change.

**Fig 5:**
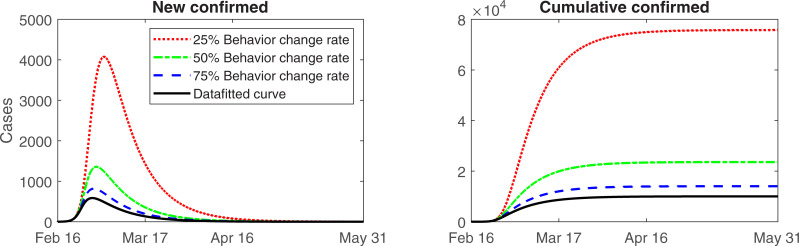
Expected number of cases as the public behavior change measures are reduced. Daily (left) and cumulative (right) number of confirmed cases.

Our findings suggest that behavior changes and social distancing measures are critical to mitigating the spread of this epidemic (Fig 5). However, these measures cannot eliminate the disease. Recently, the United States Centers for Disease Control and Prevention (CDC) has warned of the risk of a second wave of COVID-19 in the late fall/early winter 2020. The CDC director announced that social distancing will be implemented to mitigate the disease spread [11]. The spokesperson for the White House Coronavirus Task Force has also indicated that COVID-19 may return with a second wave during October-November period [12]. Mathematical modeling might help assess the risk of a second wave. Once new confirmed cases become sporadic and social distancing is reduced, outbreak reoccurrence is possible even without an imported case; concurrently, an imported case of COVID-19 might exacerbate the second wave.

The present study findings indicate that susceptible individuals should remain vigilant about the possibility of outbreak reoccurrence and maintain a high level of personal hygiene. Concurrently, governments should develop guidelines on sustainable social distancing that can mitigate the second wave of this epidemic at a lower socio-economic cost. Second, antiviral drugs and vaccine need to be made available as soon as possible, as pharmaceutical interventions are critical to ending this epidemic.

The present study has several limitations and was based on a set of assumptions that should be considered when interpreting its findings. The model included parameters based on data from the early phase of the epidemic; while some parameters which have not studied so far such as transmission reduction factor of behavior-changed individuals are assumed. As no reliable data are available on the number of asymptomatic cases or the associated transmissibility, unreported asymptomatic cases were not considered in this model, while reported asymptomatic cases were assumed to have the same transmissibility as the symptomatic cases. In Korea, we considered that the transmission possibility from exposed or asymptomatic infectious is not relatively high because of the intensive contact tracing system. All close contacts with confirmed cases need to be tested regardless of the symptoms. Even if the confirmation test of close contact were negative, they need to follow 14 days self-quarantine policy and they are released from the quarantine after additional test at the end of the quarantine. Moreover, the government emphasized to wear a facial mask in public space and keep their personal hygiene. Among the confirmed cases, over 90% of cases were identifiable the epidemic link of transmission. It also supports that transmitted cases from exposed or asymptomatic cases beyond the government monitoring were not relatively high. The cases confirmed at the airport were excluded from model of local transmission dynamics. Once the exposed individuals entered the country and became infected, they were regarded as locally transmitted cases, based on the KCDC data classification system. These considerations might have resulted in overestimation of the parameters.

In conclusion, social distancing is effective at curbing the epidemic of an emerging infectious disease, for which pharmaceutical interventions are unavailable. Proactive participation of the general public and suitable government policies are essential. Susceptible individuals need to be conscious of the disease risk and adhere to the relevant prevention guidelines. However, COVID-19 cannot be eliminated with non-pharmaceutical measures alone. Long-term social distancing is associated with high socio-economic cost. Availability of vaccine and antiviral drugs is paramount to achieving the end of COVID-19 pandemic. Further research should determine how to balance non-pharmaceutical and pharmaceutical interventions.

## Supporting information

S1 File Details of the mathematical model. (DOCX)

## Abbreviations

ROK: Republic of Korea
COVID-19: Coronavirus Disease-2019
WHO: World Health Organization
KCDC: Korea Centers for Disease Control and Prevention
CDC: United States Centers for Disease Control and Prevention
