# Supplementary material for: The impact of social distancing and public behavior changes on COVID-19 transmission dynamics in the Republic of Korea
Source: PLoS One. 2020 Sep 24;15(9):e0238684. doi: 10.1371/journal.pone.0238684 (PMC7514094; doi:10.1371/journal.pone.0238684)
Supplement: S1 File — (DOCX) [file pone.0238684.s001.docx]

**Mathematical model of COVID-19 transmission dynamics**

The total population is classified into six heterogenic classes: susceptible ($S$), behavior-changed susceptible ($S_{F}$), exposed ($E$), infectious ($I$), confirmed and isolated ($Q$), and recovered ($R$) individuals. Mathematical model consists of changing rate of each class as systems of ordinary differential equations. Figure 1 displays a flow diagram of COVID-19 transmission dynamics.


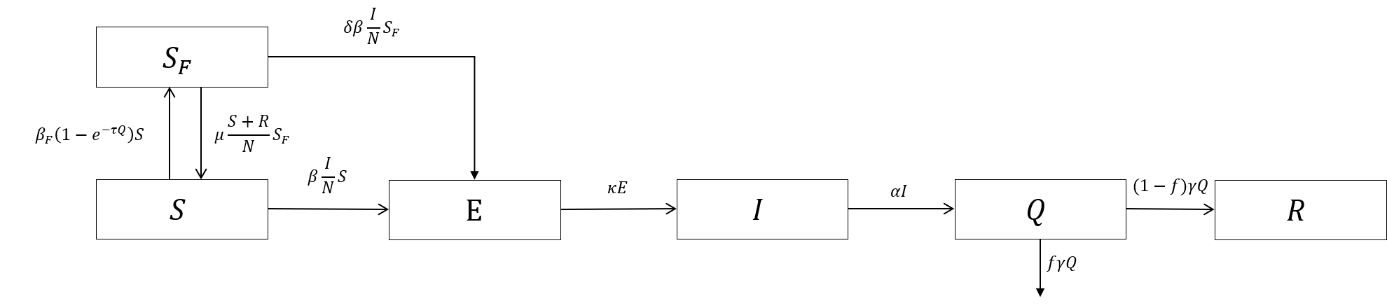


Figure 1 Transmission dynamics of COVID-19

Susceptible individuals are exposed to the COVID-19 via close contact with infectious individuals. With assumption of frequency dependent transmission, transmission term is written as $\beta\frac{I}{N}S$ with the transmission rate $\beta$. After the incubation period, the exposed individuals become infectious. We assume that every infectious individual is isolated and isolated individuals will recover or die. As isolated individuals increased, susceptible individuals become behavior-changed susceptible with a rate of $\beta_{F}\left( 1-e^{-\tau Q} \right)$. The behavior change rate is presented as $\beta_{F}$ and characteristic number is $\tau$ which adjusts scale and dimension of the model. These behavior-changed individuals have lesser susceptibility with reduction factor $\delta$. These behavior-changed susceptible individuals become susceptible again as they witness appearance of recovered individuals and other susceptible individuals. This phenomenon can be formed similarly as the transmission term, $\mu\frac{S+R}{N}S_{F}$. This COVID-19 transmission dynamics model equations are formulated as follows,

$$\frac{dS}{dt}=-\beta\frac{I}{N}S-\beta_{F}\left( 1-e^{-\tau Q} \right)S+\mu\frac{S+R}{N}S_{F} ,$$

$$\frac{dS_{F}}{dt}=\beta_{F}\left( 1-e^{-\tau Q} \right)S-\delta\beta\frac{I}{N}S_{F}-\mu\frac{S+R}{N}S_{F},$$

$$\frac{dE}{dt}=\beta\frac{I}{N}S+\delta\beta\frac{I}{N}S_{F}-\kappa E,$$

$$\frac{dI}{dt}=\kappa E-\alpha I,$$

$$\frac{dQ}{dt}=\alpha I-\gamma Q,$$

$$\frac{dR}{dt}=(1-f)\gamma Q,$$

$$N=S+S_{F}+E+I+Q+R,$$

The description and values of the parameters are listed in the following table.

| Symbol | Description | Value |
| --- | --- | --- |
| $\beta$ | The transmission rate of COVID-19 disease | 4.6180 |
| $\beta_{F}$ | The transmission rate of the awareness/fear of the disease | 2.6044 |
| $\mu$ | Behavior change eased rate | 1/14 |
| $1/\tau$ | The characteristic number of confirmed individuals reported by the news | 1000 |
| $\delta$ | The transmission reduction ratio of behavioral changed individuals | 0.02 |
| $\kappa$ | Progression rate | 1/4.1 |
| $\alpha$ | Isolation rate | 1/4 |
| $\gamma$ | Recovery rate | 1/14 |
| $f$ | Fatality rate | 0.0221 |
